# Disruptions in primary visual cortex physiology and function in a mouse model of Timothy syndrome

**DOI:** 10.1093/cercor/bhaf162

**Published:** 2025-06-27

**Authors:** Rosie Craddock, Cezar M Tigaret, Frank Sengpiel

**Affiliations:** School of Biosciences, Cardiff University, Museum Avenue, Cardiff CF10 3AX, United Kingdom; Neuroscience and Mental Health Innovation Institute, Maindy Road, Cardiff CF24 4HQ, United Kingdom; Neuroscience and Mental Health Innovation Institute, Maindy Road, Cardiff CF24 4HQ, United Kingdom; School of Biosciences, Cardiff University, Museum Avenue, Cardiff CF10 3AX, United Kingdom; Neuroscience and Mental Health Innovation Institute, Maindy Road, Cardiff CF24 4HQ, United Kingdom

**Keywords:** action potential, contrast sensitivity, mouse, Timothy syndrome, visual cortex

## Abstract

Timothy syndrome (TS) is a rare genetic disorder caused by mutations in the *CACNA1C* gene, which encodes the L-type calcium channel α_1_ Ca_V_1.2 subunit. While it is expressed throughout the body, the most serious symptoms are cardiac and neurological. Classical TS type 1 (TS1) and TS type 2 (TS2) mutations cause prolonged action potentials (APs) in cardiomyocytes and in induced neurons derived from pluripotent stem cells taken from TS patients, but the effects of TS mutations on neuronal function in vivo are not fully understood. TS is frequently associated with autistic traits, which in turn have been linked to altered sensory processing. Using the TS2-neo mouse model, we analyzed the effects of TS2 mutation on the visual system. We observed a widening of APs of pyramidal cells in ex vivo patch clamp recordings and an increase in the density of parvalbumin-positive cells in the primary visual cortex. Neurons from TS2-neo mice recorded extracellularly in vivo were less likely to respond to visual stimuli of low spatial frequency, but more likely to respond to visual stimuli of mid-to-high spatial frequency, compared to those from wild-type mice. These results point to a basic processing abnormality in the visual cortex of TS2-neo mice.

## Introduction

Timothy syndrome (TS) is a rare, multisystemic, genetic condition that is associated with a combination of cardiac (long QT interval and arrhythmias), endocrine (pancreatic, adrenal, and thyroid dysfunction), and neuropsychiatric manifestations and a somatic morphological phenotype (cardiovascular malformations and syndactyly). Neurological symptoms of TS include autism spectrum disorder (ASD), epilepsy, and developmental delay (Splawski et al. 2004; Splawski et al. 2005; Walsh et al. 2018). TS is caused by mutations in the *CACNA1C* gene, which is associated with risk across a number of neuropsychiatric conditions (Liu et al. 2011; Lu et al. 2012). *CACNA1C* encodes the α_1_ Ca_V_1.2 subunit of L-type voltage-gated calcium channels (L-VGCCs), which are expressed in a variety of tissues (Uhlén et al. 2015).

Subtypes of TS are typically categorized by causative mutation in the *CACNA1C* gene. Two missense G406R point mutations in exons 8A and 8 cause, respectively, TS type 1 (TS1) and TS type 2 (TS2) (Splawski et al. 2004, 2005; Bauer et al. 2021). L-type Ca^2+^ channels containing an α subunit with TS mutations TS voltage-gated calcium channels (TS-VGCCs) have a reduced voltage-dependent inactivation (Barrett and Tsien 2008). Consequently, TS-VGCCs remain open for a prolonged period upon channel activation, generating prolonged depolarizing calcium influx into the cell (Splawski et al. 2004, 2005; Paşca et al. 2011; Yazawa et al. 2011). In cardiomyocytes, the functional properties of TS-VGCCs result in an increased duration of cardiac action potentials (APs), thought to underlie cardiac symptoms in TS, including long QT, arrhythmia, and cardiac arrest (Splawski et al. 2004, 2005; Dick et al. 2016). By contrast, the mechanistic links between TS-VGCCs and the neurological phenotype in TS are not yet understood.

In neurons, Ca_V_1.2 L-VGCCs are expressed predominantly somatodendritically and are activated by membrane depolarization during APs to generate Ca^2+^ influx important for synaptic function, shaping of AP firing, and regulation of gene expression (Zamponi et al. 2015). In addition, L-VGCCs have been critically implicated in neurodevelopmental neuronal migration (Jiang and Swann 2005; Kamijo et al. 2018). Observations in neurons differentiated from TS patient–derived induced pluripotent stem cells (iPSCs) indicate that the neurobiological consequences of TS mutation are electrophysiological (prolongation of APs and increase of residual cellular Ca^2+^; Paşca et al. 2011) and neurodevelopmental (aberrant interneuron migration; Birey et al. 2017). This suggests that TS and other neurodevelopmental conditions have a neurodevelopmental pathology in common (Hussman 2001; Paterno et al. 2021a; Righes et al. 2021). However, the impact of TS-VGCCs on neuronal and circuit functional properties in the adult brain is less clear.

Moreover, abnormalities in inhibitory interneuron migration have been demonstrated in cultured human forebrain spheroids from TS1 patients (Birey et al. 2017). The development of parvalbumin-positive (PV+) cells, the most numerous type of inhibitory interneurons, is regulated by L-VGCCs, including Ca_V_1.2 (Jiang and Swann 2005). Therefore, functional changes in the channel resulting from TS mutations could impact PV+ cell development and migration.

Abnormalities in visual perception and in vision-related behaviors have been reported in some TS patients (Gillis et al. 2012; Bozarth et al. 2018). These sensory processing abnormalities are known to be associated with ASD, which is also frequently exhibited in TS patients. Both gaze aversion and the limited interest in faces displayed by ASD patients have been attributed to abnormal processing of facial visual stimuli (Dalton et al. 2005; Coulter 2009; Zhou et al. 2023). It has been proposed that the lack of visual attention to facial stimuli and reduced ability to perceive and recognize faces in ASD patients occur as a result of differences in basic visual processing in the ASD brain (Coulter 2009; Vlamings et al. 2010; Zhou et al. 2023), including enhanced contrast sensitivity for simple visual grating stimuli of high spatial frequency (SF; Kéïta et al. 2014) and increased ability to see the fine detail in visual images, as opposed to the big picture (Shah and Frith 1983; Robertson and Baron-Cohen 2017; Zhou et al. 2023). However, the mechanistic roles of TS-VGCCs in this sensory phenotype are not well understood (Marco et al. 2011).

In order to understand the consequences of TS-VGCCs at the neuronal and systems levels on sensory processing in the adult brain, we used a mouse model expressing the TS2 mutation (TS2-neo mouse) exhibiting TS-relevant behavioral and neurodevelopmental abnormalities (Bader et al. 2011a; Bett et al. 2012; Rendall et al. 2017; Horigane et al. 2020). TS2-neo mice have a superior ability to perceive complex auditory stimuli (Rendall et al. 2017), without gross perceptual disturbances (Bett et al. 2012), consistent with the perceptual enhancements reported in ASD patients (Bertone et al. 2005; Mottron et al. 2006). However, the links between TS2 mutations and visual processing abnormalities are less clear.

Here, we describe a series of experiments using TS2-neo mice aimed to explore the neurobiological consequences of the TS-VGCCs on visual processing in the primary visual cortex (V1), AP firing properties of V1 pyramidal neurons, and the density of PV+ interneurons in the visual cortex.

## Materials and methods

### Animals

All the experimental procedures were carried out in accordance with the UK Animals (Scientific Procedures) Act 1986 and European Commission Directive 2010/63/EU. Mice heterozygous for the TS-type TS2-neo mutation (background C57BL/6J) were purchased from Jackson Labs (Jax #019547) and bred with C57BL/6J mice also purchased from Jackson Labs (Jax #000664). Mice generated from this breeding colony were used in all the experiments described here. Same-sex littermates were housed together under normal light conditions (12-h light and 12-h dark) and were given standard food and water ad libitum. Environmental enrichment was provided to the mice. The experimenters were kept blind to the genotype throughout the study until completion of final data analyses in each instance. Animal treatments and usage specific to each experiment are detailed at the beginning of the following subsections.

### Electrophysiology

In this experiment, 8 mice heterozygous for the TS2-neo mutation and 5 sibling wild-type (WT) mice of both sexes were used. Mice aged 4 to 10 weeks old were sacrificed by an overdose of isoflurane before decapitation and rapid removal of the brain under ice-cold choline chloride slicing solution (110 mM choline chloride, 25 mM d-glucose, 15 mM NaHCO_3_, 2.5 mM KCl, 1.25 mM NaH_2_PO_4_, 11.6 mM l-ascorbic acid, 2.1 mM sodium pyruvate, 0.5 mM CaCl_2_, 7 mM MgCl_2_, and 310 mOsm). Brains were coronally sectioned to a thickness of 400 μm using a vibratome (CI.7000SMZ-2, Campden Instruments). Sections containing V1 were collected and incubated for 30 min in carboxygenated (95% O_2_, 5% CO_2_) artificial cerebrospinal fluid (ACSF; 11.9 mM NaCl, 10 mM d-glucose, 2.6 mM NaHCO_3_, 2.5 mM KCl, 1 mM NaH_2_PO_4_, 2.5 mM CaCl_2_, and 1.3 mM MgCl_2_) at 34°C in a brain slice holding chamber (BSK1, Brain Slice Keeper). After incubation, the brain slice holding chamber was left at room temperature for at least 1 h before use.

Slices were mounted onto poly-l-lysine-coated glass coverslips, which were then placed into a submerged recording chamber (Slice Minichamber II, Luigs and Neumann), which was perfused with carboxygenated ACSF at 32°C. Pyramidal cells in V1 were visualized using an infrared differential interference contrast imaging system (BX51 WT, Olympus). Putative pyramidal cells were targeted based on somatic shape, size, and location in layers 2 to 5 of V1. The cell type was later confirmed by biocytin–streptavidin histology and confocal imaging using a confocal microscope (Zeiss Airyscan LSM880). A maximum of two cells were recorded from each animal.

Micropipettes filled with internal solution (117 mM potassium methanesulfonate, 8 mM NaCl, 10 mM 4-(2-hydroxyethyl)-1-piperazineethanesulfonic acid, 4 mM magnesium adenosine triphosphate (MgATP), 0.3 mM sodium guanosine triphosphate (NaGTP), and 0.2 mM ethylene glycol-bis(β-aminoethyl ether)-*N*,*N*,*N*′,*N*′-tetraacetic acid, pH 7.4, 280 mOsm) were used to attain whole-cell patch clamp. The pipette resistance was measured at 4 to 5 MΩ in each instance. The liquid junction potential was corrected for using MultiClamp 700B software. Patched cells were held at −70 mV for 1 min before measuring membrane potential responses to a series of incremental current step injections between −50 and 650 pA. The duration of each current step injection was 200 ms, followed by a 2 s 0 pA injection; the increment between steps was 50 pA. The experimental protocol was designed and implemented using Clampex 11 software. Membrane potential data were low-pass filtered to 6 kHz, sampled at 50 kHz, and digitized using an Axon Digidata 1550 Data Acquisition System and Clampex 11 software.

Analysis of the electrophysiological recordings was performed with custom software written using Python 3.10 and the pyABF package (Harden 2022). The recorded membrane potential trace resulting from the hyperpolarizing −50 pA current step injection was used to measure the passive membrane properties of the cell. A more detailed description of how the passive membrane potential properties were measured is provided in the Supplementary Methods and in Fig. S1. Measurements of single AP properties were taken from the first AP, which was fired during the experimental protocol. The membrane potential threshold was measured as the membrane potential at which the rate of change of the membrane potential reached 15.2 mV/ms (Paşca et al. 2011). The membrane potential halfway between the threshold potential and the AP amplitude was designated as the AP half-height. The duration of time for which the membrane potential exceeded this AP half-height for the first measured AP was taken as the AP half-width. The total AP rise and fall times were taken as the time between threshold and amplitude, and amplitude to return to threshold potential, respectively.

Data analysis was completed in R. The distributions of all the properties within the dataset for V1 cells were tested for normality by Shapiro–Wilk testing. Membrane capacitance, time constant, threshold potential, AP amplitude, AP half-width, maximum rates of rise and fall of the AP, total time for AP rise and fall were all found to be normally distributed by Shapiro–Wilk testing (*P* > 0.05), while the values of membrane input resistance in the dataset were found to be non-normally distributed by Shapiro–Wilk testing (*P* = 0.038, *W* = 0.88). Genotype differences in all normally distributed variables were tested by *t*-tests, while the genotype difference in membrane input resistance was tested by the Wilcoxon signed-rank test.

### In vivo two-photon calcium imaging of V1

Data for this study were obtained from 5 TS2-neo and 4 WT mice of either sex. Mice underwent surgery at 10 to 12 weeks of age. Surgical procedures were based on those described previously by our lab (Powell et al. 2020; Craddock et al. 2023). Mice were given prophylactic antibiotics and anti-inflammatory medication for 1 week prior to surgery. Mice were anesthetized using isoflurane and secured in a stereotaxic frame (David Kopf Instruments, Tujunga, CA, United States). The scalp and periosteum were removed from the dorsal surface of the skull. A custom head plate was attached to the cranium using dental cement (Super Bond C&B). The aperture of the head plate was cantered over the right hemisphere V1 of the mouse brain, 2.3 mm lateral and 3.1 mm posterior to the bregma. A 3 mm circular craniotomy was made in this position. Mice were injected with 150 μL of AAV1.Syn.GCaMP6f.WRPE.SV40 viral vector (Penn Vector Core, 1:10 dilution of 3.45 × 10^13^ gc/mL) in V1b (3.1 mm lateral and 3.1 mm posterior to the bregma) such that the fluorescent calcium indicator, GCaMP6f, was expressed in neurons of V1b of the mouse brain. The craniotomy was closed using 2 × 3 mm circular glass inserts bonded together, overlaid with a 5 mm circular insert (Biochrom Ltd.; 3 mm: product code 640720[CS-3R]; 5 mm: product code 640700), which was attached to the cranium-fixed head plate. Glass inserts were bonded together using optical adhesive (Norland Products; catalog no. 7106). The window was sealed with dental cement and the mice left to recover under surveillance. Mice were returned to their home cage with sibling mice and left to recover for 1 week. The mice received postoperative checkups for the first 3 d of this recovery period and were administered postoperative anti-inflammatories and antibiotics.

Mice underwent a 1-week period during which they were habituated to handling and head fixation using methods similar to those described by Aguillon et al. (2021). Mice were placed on top of a custom-built cylindrical treadmill and were head-fixed to a resonant scanning two-photon microscope (Thorlabs, B scope) by the mouse’s cranium-mounted head plate. Mice were positioned to face a calibrated LCD PC screen (iiyama, B2080HS; width × height 26 × 47 cm^2^) positioned in the mouse’s binocular field of view, 20 cm away from the mouse.

In vivo two-photon imaging was then performed on awake mice receiving visual stimulation via the screen positioned in their binocular field of view. Imaging involved a resonant scanning two-photon microscope equipped with a 16× and 0.8 NA water-immersion objective (Nikon). GCaMP6f in neurons of L2/3 was excited at 920 nm using a Ti:sapphire laser (Coherent, Chameleon) using a maximum power of 50 mW (as measured at the sample). Data were acquired using ThorImage software at a raw frame rate of 56 Hz. We averaged over six frames, giving an effective frame rate of 9.6 Hz. Imaging timestamps were collected using MATLAB 2022a and ThorSync software and National Instruments data acquisition cards. Recordings were made from a 1250 × 1250 μm (256 × 256 pixel) field of view, at a depth of 150 to 250 μm from the pial surface, corresponding to cortical L2/3 of V1b. Each mouse was recorded up to 3 times, with up to two recordings being taken from a single mouse in 1 d. Each of the three recordings was taken from a different field of view within V1b.

Visual stimuli were designed and generated using custom-made codes written in MATLAB (2022a) and in C# alongside the Psychophysics toolbox (Brainard 1997). Stimuli were presented on the calibrated LCD PC screen described previously. The codes used to run the experiment are available on GitHub. The software ThorImage and ThorSync are open source and are available on request from Thorlabs. Visual stimuli were unidirectionally drifting (to the mouse’s right) vertical sinusoidal gratings shown across the full monitor screen. The drifting gratings had a temporal frequency of 1.25 Hz and varied in SF and in contrast. Stimuli of seven different SFs were shown to each mouse: 0.014 , 0.031, 0.064, 0.128, 0.236, and 0.383 cycles per degree (cpd). The stimuli of each SF tested were shown at 100%, 50%, 25%, 12.5%, 6.7%, and 3.4% contrast such that we could find the minimum contrast at which the mouse’s V1b pyramidal neurons responded to the visual stimuli of each SF tested. The contrasts and SFs used in this experiment were based on those used in similar experiments previously (Prusky and Douglas 2004; Umino et al. 2018; Cheng et al. 2020). In the experiment, 42 different visual stimuli were used—one for each SF at each contrast. These were shown in a pseudo-random order to the mouse, and this was repeated 5 times in a single experimental recording. Single visual stimuli were shown for 3 s, followed by a 3 s baseline period where a gray screen of equivalent luminescence was shown to the mouse.

Images were registered and cells detected using suite2P software (Pachitariu et al. 2017). Further details are described in the Supplementary Methods.

Further analyses were conducted in R. The proportion of all the cells that were found to be visually responsive was compared by genotype using Wilson’s test of equal proportions (Wilson 1927). Visually responsive cells were sorted into categories of contrast sensitivity for stimuli of each SF, where contrast sensitivity is defined as $\mathrm{contrast}\ \mathrm{sensitivity}=\log 2\left(\frac{1}{\mathrm{minimum}\ \mathrm{contrast}\ \mathrm{required}\ \mathrm{to}\ \mathrm{elicit}\ \mathrm{a}\ \mathrm{response}}\right)$. Cells that were nonresponsive to visual stimuli of a specific SF were placed into the contrast sensitivity category “NR”; for details, see Supplementary Methods. Post hoc tests of the effect of the genotype on the contrast sensitivity at each SF were completed using the emmeans and multcomp packages in R (Searle et al. 1980; Hothorn et al. 2023).

### Histology, immunofluorescence techniques, and imaging

Tissues from 7 mice heterozygous for the TS2-neo mutation and from 4 WT mice were used to obtain PV+ cell counts from V1 in this experiment. Mice were of either sex and were 20 weeks of age at the time of sacrifice and tissue extraction. Mice were anesthetized using isoflurane and sacrificed using an overdose of intraperitoneal Euthatal. Mice were then transcardially perfused with 4% paraformaldehyde (PFA) in phosphate-buffered saline (1× PBS) solution. Whole brains were extracted and stored in 4% PFA for 48 h before being washed and stored in 1× PBS for 5 d. Brains were sectioned coronally to 50 μm using a Leica LS1000 vibratome. One in four sections containing V1 were collected and stored in 1× PBS for up to 56 h. All the washes described in this protocol were 10 min long and were completed at room temperature. Sections were washed 3 times in 1× PBS, and then washed a further 4 times in 1× PBS containing 0.2% triton. Sections were incubated in a blocking solution (3% normal goat serum in 1× PBS with 0.2% triton) for 1 h at room temperature. Sections were then incubated with primary antibody solution (1:1,000 rabbit anti-PV [Swant, PV27], 3% normal goat serum in 1× PBS with 0.2% triton) for 48 h at 4°C. Sections were washed 3 times in 1× PBS with 0.2% triton. Sections were incubated in secondary antibody solution (1:500 goat anti-rabbit Alexa Fluor 488 [Invitrogen, A-11008] in 1× PBS with 0.2% triton) for 2 h at room temperature. Sections were washed 3 times in 1× PBS with 0.2% triton and then stained with Hoechst 33342 solution to provide a control nuclear stain. Sections were washed 4 times in 1× PBS with 0.2% triton before being mounted onto SuperFrost Plus microscope slides and coverslipped using Fluoromount aqueous mounting medium. The coverslip edges were sealed using clear nail polish.

Images were obtained using an Olympus VS200 ASW slide scanner at 4× magnification. Images were taken at one depth per section only. This depth of focus for imaging was selected automatically by the autofocus feature of the Olympus VS2000 ASW microscope and associated OlyVIA software. PV+ cell counts were collected using automated methods in FIJI software. Cell counts were taken respective to the area of V1 within the imaged section to obtain a measurement of PV+ cell density per millimeter squared. At least eight sections were used to obtain V1 PV+ cell density measurements from each animal (range: 8 to 26 sections per animal).For the statistical analysis of PV+ cell density data, see Supplementary Methods.

### Correction for multiple testing and reporting results

There were three primary hypotheses in this study: that TS2-neo heterozygosity would (i) prolong the AP in pyramidal cells of V1, (ii) alter the contrast sensitivity recorded in mice, and (iii) alter the PV+ cell density within V1. The three *P* values related to the three primary hypotheses were corrected using the stringent Bonferroni correction method.

In this series of experiments, 12 tests related to the secondary hypotheses of this study were completed. *P* values from these tests were corrected for 12 multiple tests by use of the Benjamini–Hochberg method.

All the test values, *P* values, estimated effect sizes, and summary values are given to two significant figures in this article. Where summary values are given, the mean value is provided alongside the standard deviation in that value, the number of observations is given as *n*, and the number of animals is given as *N*.

## Results

### Prolonged AP in V1 pyramidal cells of the TS2-neo mice

To understand the electrophysiological consequences of TS-VGCCs in the visual cortex, we measured passive membrane and AP firing properties of V1 pyramidal neurons in ex vivo slices, in whole-cell patch clamp recordings. All the data reported here are from 25 cells recorded in 8 mice heterozygous for the TS2-neo mutation and 11 cells recorded in 5 sibling WT mice.

Passive membrane properties (see Fig. S1), including the membrane input resistance (*R*_in_), membrane capacitance (cap), and membrane time constant (*τ*) were not found to be impacted by genotype: The median input resistance was 86.80 MΩ (interquartile range [IQR] 58.06 to 134.9 MΩ) in TS2-neo mice and 104.2 MΩ (IQR 68.22 to 127.4) in WT mice, while the membrane capacitance was 183.6 ± 126.2 pF (mean ± SD) in TS2-neo mice and 151.8 ± 70.21 in WT mice (statistics given in Table 1). The AP threshold and amplitude also did not vary significantly by genotype. The AP threshold was −43.97 ± 5.323 mV in TS2-neo mice and −44.55 ± 3.905 mV in WT mice, while the AP amplitude was 65.06 ± 9.163 mV in TS2-neo mice and 69.56 ± 10.71 mV in WT mice (see Table 1). Furthermore, there was no difference in rheobase between TS2-neo mice (median, 150.2 pA; IQR = 100.2 to 250.4) and WT mice (median, 150.2 pA, IQR = 149.9 to 200. 1).

**Table 1:** Electrophysiological parameters of V1 pyramidal cells in TS2-neo mice and wild-type (WT) mice.^a^

Parameter	TS2-neo		WT		Statistics TS2-neo vs WT
Membrane capacitance (pF)	Mean = 183.6 (*n* = 17)	SD = 126.2	Mean = 151.8 (*n* = 9)	SD = 70.21	*t*-test, *P* = 0.58, corr. *P* = 0.81^b^
Membrane input resistance (MΩ)	Median = 86.80 (*n* = 17)	IQR = 58.06 to 134.9	Median = 104.2 (*n* = 9)	IQR = 68.22 to 127.4	Wilcoxon, *P* = 0.72, corr. *P* = 0.81
Membrane time const. (ms)	Mean = 15.95 (*n* = 17)	SD = 11.20	Mean = 12.89	SD = 6.2333	*t*-test, *P* = 0.34, corr. *P* = 0.81
Voltage sag (mV)	Median = 0.8631 (*n* = 17)	IQR = 0.7264 to 1.711	Median = 0.9829 (*n* = 9)	IQR = 0.7956 to 1.190	Wilcoxon, *P* = 0.96, corr. *P* = 1
Membrane potential rebound (mV)	Median = 2.057 (*n* = 17)	IQR = 1.430 to 2.974	Median = 2.469	IQR = 1.359 to 4.234	Wilcoxon, *P* = 0.53, corr. *P* = 0.89
Rheobase (pA)	Median = 150.2 (*n* = 17)	IQR = 100.2 to 250.4	Median = 150.2 (*n* = 9)	IQR = 149.9 to 200. 1	Wilcoxon, *P* = 0.879, corr. *P* = 1
AP threshold (mV)	Mean = −43.97 (*n* = 25)	SD = 5.32	Mean = −44.55 (*n* = 11)	SD = 3.91	*t*-test, *P* = 0.81, corr. *P* = 0. 89
AP amplitude (mV)	Mean = 65.06 (*n* = 25)	SD = 9.16	Mean = 69.6 (*n* = 11)	SD = 10.7	*t*-test, *P* = 0.65, corr. *P* = 0.81
AP half-width (ms)	Mean = 0.978 (*n* = 25)	SD = 0.184	Mean = 0.826 (*n* = 11)	SD = 0.243	*t*-test, ***P* = 0.016, corr. *P* = 0.048**
AP max rate of rise	Mean = 227,203 (*n* = 25)	SD = 69,505	Mean = 276,600 (*n* = 11)	SD = 64,849	*t*-test, *P* = 0.051, corr. *P* = 0.31
AP total rise time (ms)	Mean = 0.6008 (*n* = 25)	SD = 0.09975	Mean = 0.5382 (*n* = 11)	SD = 0.1137	*t*-test, ***P* = 0.032**, corr. *P* = 0.22
AP max rate of fall	Mean = 71,655 (*n* = 25)	SD = 18,221	Mean = 92,246 (*n* = 11)	SD = 30,907	*t*-test, ***P* = 0.023**, corr. *P* = 0.20
AP total fall time (ms)	Mean = 1.908 (*n* = 23)	SD = 0.9186	Mean = 1.385 (*n* = 11)	SD = 0.4679	*t*-test, ***P* = 0.025**, corr. *P* = 0.20

^a^Where data were normally distributed mean and standard deviation (SD) are given; where data were not normally distributed median and interquartile range (IQR) are given.

^b^ *P* values are given before and after correction for multiple comparisons (corr.).

Significant differences (*P* < 0.05) are highlighted in bold.

**Fig. 1:**
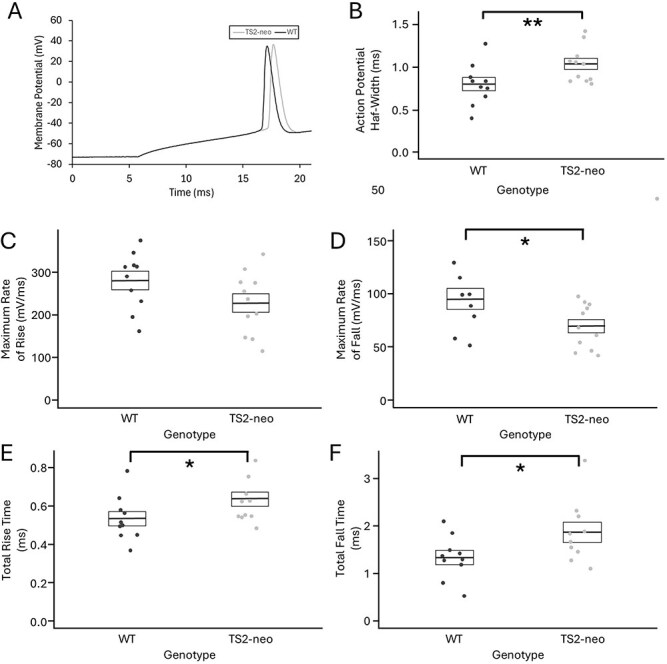
The TS2-neo mutation causes an increase in AP width. A) Representative AP waveforms from a V1 pyramidal cell of a TS2-neo mouse (gray line) and a WT mouse (black line). B) AP half-widths measured in V1 pyramidal cells of TS2-neo mice (right) and WT mice (left). Box plots show mean ± SEM, dots show values for each individual cell (WT: *n* = 10; TS2-neo: *n* = 11). ^**^ indicates significant difference (*P* < 0.05) both before and after correction for multiple comparisons. AP maximum rate of C) rise and D) fall, E) total rise time and F) total fall time compared for V1 pyramidal cells in TS2-neo mice and WT mice. Box plots show mean ± SEM, dots show values for each individual cell (WT: *n* = 10; TS2-neo: *n* = 11). ^*^ indicates significant difference (*P* < 0.05), which was lost after correction for multiple comparisons (see Table 1 for individual *P* values).

The AP duration, measured by the AP half-width, was found to be significantly longer in V1 pyramidal cells from TS2-neo mice (0.9784 ± 0.1842 ms, *n* = 25 cells, *N* = 8 mice) compared to those from WT mice (0.8255 ± 0.2425 ms, 24 ms, *n* = 11 cells, *N* = 5 mice; *t*-test, *P* = 0.016, corrected *P* = 0.048). The AP duration was on average 19% longer for V1 pyramidal cells from TS2-neo mice than that for V1 pyramidal cells from WT mice, based on comparison of the raw data. An example AP waveform from a V1 pyramidal cell of a TS2-neo mouse compared to that from a WT mouse is shown in Fig. 1A. The AP half-widths measured from V1 pyramidal cells of TS2-neo and WT mice are shown in Fig. 1B.

Further testing was conducted to explore whether the mutation had more of an effect on either the rising or the falling phase of the AP waveform. This provided no evidence to suggest that TS2-neo heterozygosity impacts AP rise more than it does repolarization of the membrane, or vice versa. Genotype had a significant effect on the maximum rate of fall of the AP as well as on the total duration of AP rise and fall, while the effect on the maximum rate of rise of the AP was not quite significant. However, after multiple test corrections none of these effects remained significant (see Table 1). The values obtained for each of these properties from TS2-neo and WT mice are shown in Fig. 1C to F and Table 1.

### Differences in neuronally measured contrast sensitivity functions from TS2-neo and WT mice

**Fig. 2:**
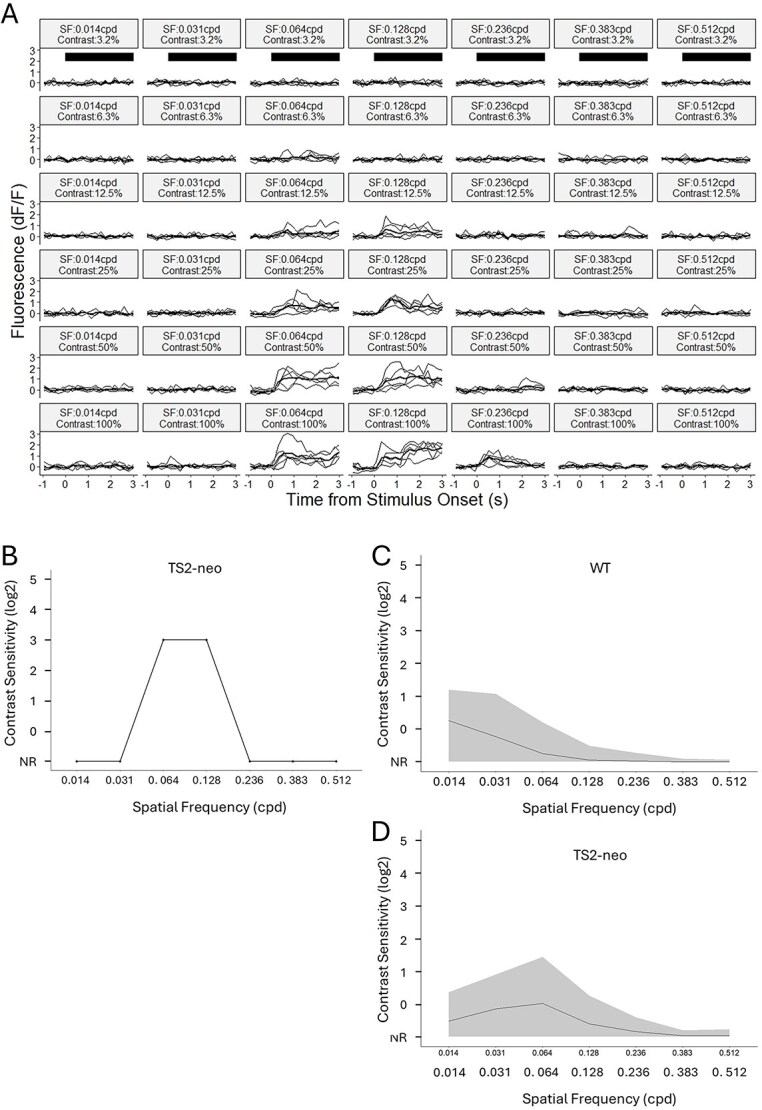
CSFs in TS2neo and WT mice. A) Fluorescence traces from a visually responsive V1 neuron of a TS2-neo mouse in response to grating stimuli of 7 different SFs and 6 contrast levels tested. Horizontal black bars in the top row (3.2% contrast) indicate the duration of the visual stimulus (3 s). B) CSF of the V1 neuron shown in (A); for details see Methods. C) Population CSF for one WT animal. The line represents the mean, the ribbon represent the IQR; for details see Supplementary Methods. D) Population CSF for one TS2-neo animal. The line represents the mean, the ribbon represent the IQR.

**Fig. 3:**
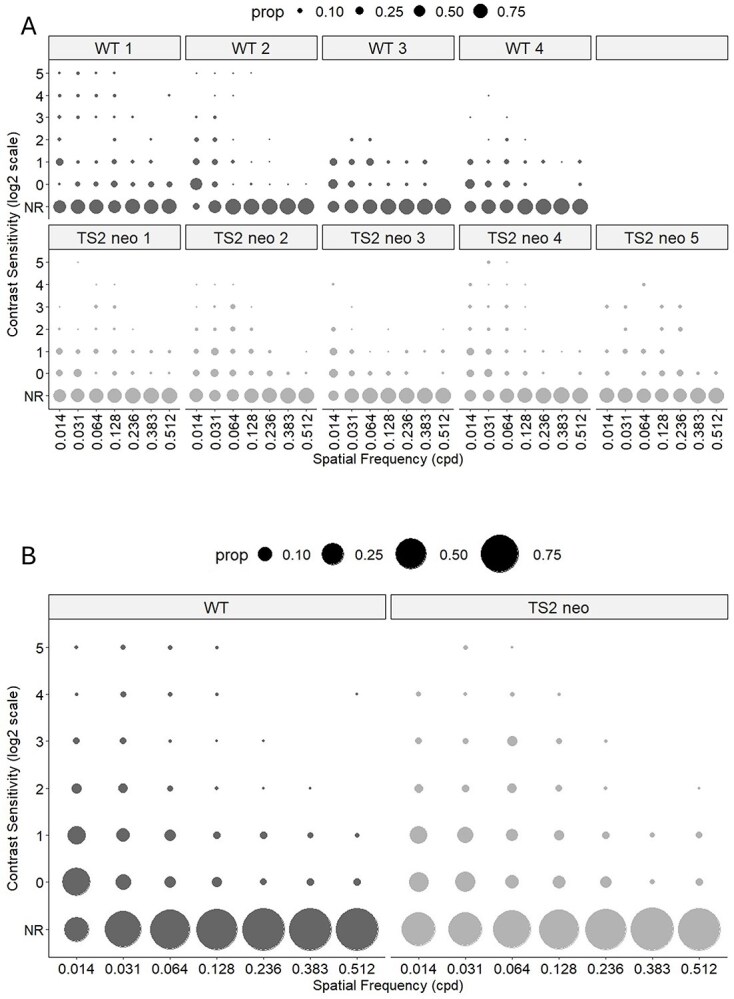
Contrast sensitivity of all the recorded V1 neurons for TS2-neo and WT mice. A) Contrast sensitivity for each individual of the 4 WT mice (top row) and 5 TS2-neo mice (bottom row). B) Contrast sensitivity of V1 neurons across all 4 WT mice (*n* = 3,360 cells, left) and all 5 TS2-neo mice (*n* = 3,990 cells, right). Circle sizes represent proportions of cells responding at the contrast sensitivities given on the ordinate for each of the spatial frequencies shown on the abscissa. Circle sizes for proportions (“prop”) of 10%, 25%, 50%, and 75%, respectively, are shown at the top. “NR” indicates visually nonresponsive neurons.

In order to assess the effect of the TS2 mutation on basic visual stimulus processing, we measured contrast sensitivity functions (CSFs) of V1b neurons using two-photon calcium imaging. WT data were obtained from 3,360 cells across 4 animals (*N* = 4, *n* = 3,360), and TS2-neo data were obtained from 3,990 cells across 5 animals (*N* = 5, *n* = 3,990). Visually responsive cells were detected as described in the Supplementary Methods section (see also Fig. S2, showing visually responsive GCaMP6f-labeled neurons responding to a stimulus of low SF [0.014 cpd] and high contrast [100%]). Fluorescence traces from each visually responsive cell were processed and used to measure the cell’s responsiveness to the visual stimuli of each SF tested and at each contrast level (see Fig. 2A). For each neuron, a CSF was generated, showing how contrast sensitivity varies with SF of the gratings (see Fig. 2B). CSFs for all the cells of a TS2-neo and a WT animal are shown in Fig. 2B and C, respectively. The contrast sensitivity of all the recorded V1 neurons for each of the 5 TS2-neo and 4 WT mice is shown in Fig. 3A. Note that there are proportionally more cells responding at lower spatial frequencies in WT mice and more cells responding at medium spatial frequencies in TS2-neo mice. The contrast sensitivity of all the V1 neurons across all the WT mice (*n* = 3,360 cells) and all the TS2-neo mice (*n* = 3,990 cells) is shown in Fig. 3B.

A cumulative link mixed model was designed to assess how contrast sensitivity dependence on SF (the overall CSF of all neurons recorded from one animal) varied with genotype while accounting for between-animal variance as a random factor. The assumptions of the model were deemed to be met since the model residuals appeared normally distributed. The model was found to explain 17% of variance in contrast sensitivity measured from all the cells (Cox and Snell: pseudo-*R*^2^ = 0.17). This value is low as expected: The SF to which a V1b neuron responds varies based on the cell’s location along the anterior/posterior extent of V1 (Zhang et al. 2015); cells in this experiment were sampled across L2/3 of V1b. Model testing found that the genotype–SF interaction term varied significantly with contrast sensitivity (analysis of deviance: *χ*^2^ = 85, *P* = 4.1 × 10^−16^, corrected *P* = 1.2 × 10^−15^). This indicates that TS2-neo heterozygosity significantly affects the neuronally measured CSF of the mouse.

**Fig. 4:**
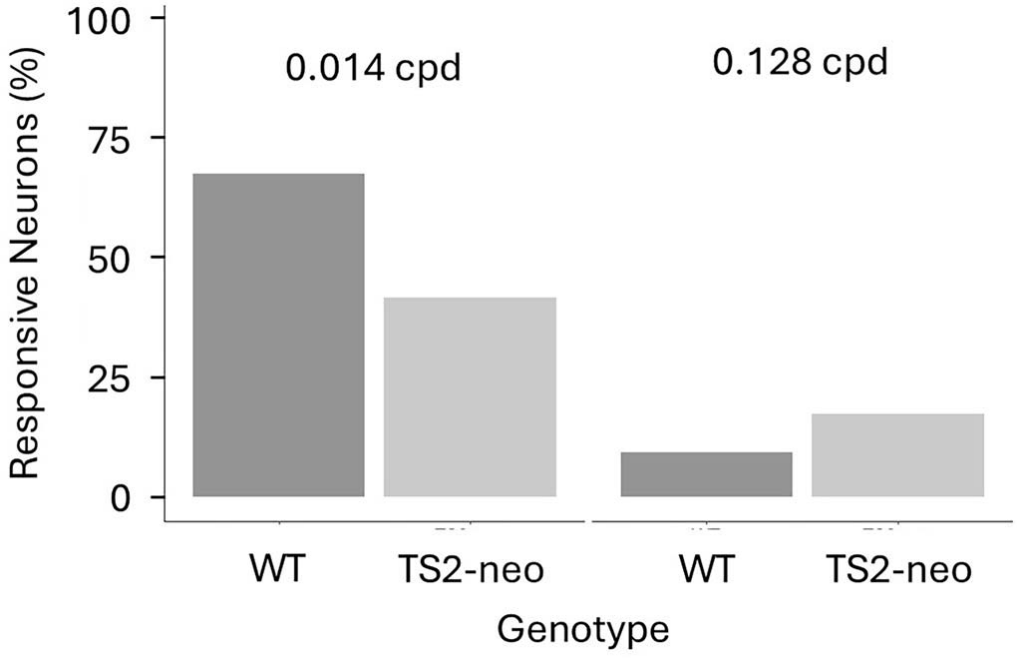
Proportions of V1b neurons from WT mice and TS2-neo mice responding to gratings of low spatial frequency (0.014 cpd, left) and proportions of V1b neurons from WT and TS2-neo mice responding to gratings of medium spatial frequency (0.128 cpd, right).

Post hoc testing was conducted to explore exactly how TS2-neo heterozygosity impacts the neuronally measured contrast sensitivity to visual stimuli of both low (0.014 cpd) and medium-to-high (0.128 cpd) SFs. TS2-neo heterozygosity was associated with a reduction in contrast sensitivity to visual stimuli of low SF (0.014 cpd [estimated marginal means]: *Z* ratio = −4.2, *P* = 2.2 × 10^−5^, corrected *P* = 2.4 × 10^−3^) and an increase in contrast sensitivity to visual stimuli of high SF, although significance was lost after correction for multiple testing (0.128 cpd [estimated marginal means]: *Z* ratio = 2.73, *P* = 0.0063, corrected *P* = 0.063). This is mirrored in the proportion of all visually responsive V1b cells from TS2-neo compared to those from WT animals which respond to high- and low-SF stimuli (Fig. 4). In WT mice, 67.3% of the neurons respond at 0.014 cpd vs 41.4% in TS2-neo mice, while conversely only 9.4% of the neurons from WT mice respond at 0.128 cpd compared to 17.4% of the neurons from TS2-neo mice.

Overall, mice heterozygous for the TS2-neo mutation have significantly different neuronally measured CSFs compared to WT mice. Neuronally measured contrast sensitivity to visual stimuli of low SF (0.014 cpd) is significantly lower in mice heterozygous for the TS2-neo mutation compared to WT mice. Neuronally measured contrast sensitivity to visual stimuli of high SF (0.128 cpd) tends to be higher in mice heterozygous for the TS2-neo mutation compared to WT mice.

### Increased PV+ cell density in V1 of TS2-neo as compared to WT mice

**Fig. 5:**
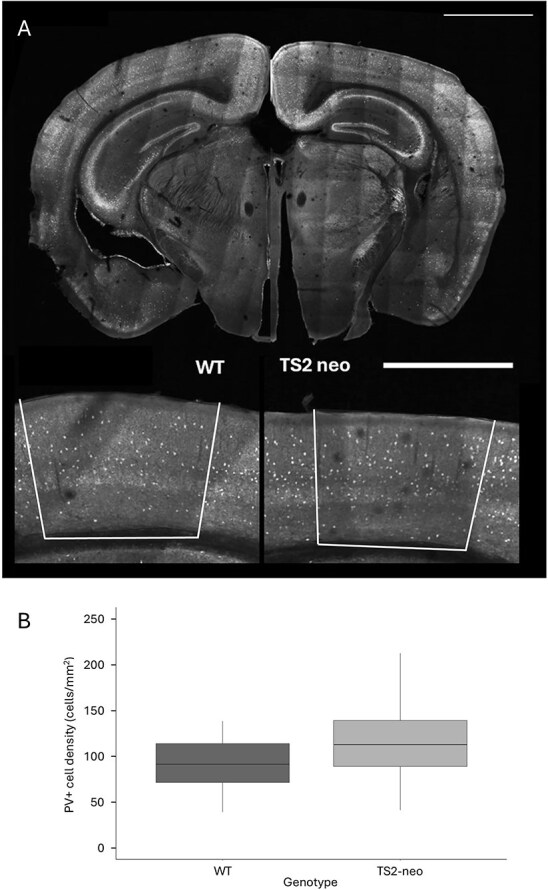
PV+ cell density is increased in V1 of TS2-neo mice compared with WT mice. A) Alexa Fluor 488–labeled PV+ cells in sections from a WT mouse (left) and TS2-neo mouse (right). The bottom images show V1 in higher magnification (boundaries outlined). Scale bars, 2 mm for the top panels and 1 mm for the bottom panels. B) PV+ cell density in V1 of WT mice (left) and TS2-neo mice (right).The boxplot shows mean ± IQR. The upper whisker (vertical line) extends from the hinge to the largest value no further than 1.5× IQR from the hinge (where IQR is the interquartile range, or distance between the first and third quartiles). The lower whisker extends from the hinge to the smallest value no further than 1.5× IQR from the hinge.

In order to ascertain whether the TS2 mutation affects the development of inhibitory interneurons in V1, we studied the distribution and density of PV+ cells, the most common GABAergic cell class in V1. Figure 5A shows Alexa Fluor 488–labeled PV+ cells in sections from TS2-neo and WT mice. The PV+ cell density values for sections collected from TS2-neo and WT mice were found to be normally distributed. Analysis of variance showed that genotype significantly affected V1 PV+ cell density (*F* = 16.81, *P* < 0.001). The PV+ cell density measurements obtained from sections of WT and TS2-neo mice are shown in Fig. 5B. TS2-neo mice had a 22.7% higher PV+ cell density in V1 as compared to WT mice (PV+ cell density in V1: WT = 93.3 ± 3.03 cells/mm^2^ [mean ± SEM; *n* = 71 sections, *N* = 4 mice]; TS2-neo = 114.9 ± 3.73 cells/mm^2^ [mean ± SEM; *n* = 106 sections, *N* = 7 mice]).

## Discussion

We found that the TS2-neo mutation prolongs APs in mouse V1 pyramidal cells, as does the TS2 mutation in cardiomyocytes. It alters basic visual processing in V1b of the mouse at the neuronal level: The CSF is characterized by decreased sensitivity to low SF and increased sensitivity to medium-to-high SF. Moreover, the PV+ cell density is increased in V1.

### Prolonged pyramidal cell AP

Our results from whole-cell patch clamp recordings in acute ex-vivo slices from TS2-neo mouse brains replicate those of Paşca et al. (2011) in human iPSC-derived neurons. They provide evidence that classical TS mutations result in a prolonged AP in neurons as they do in cardiac myocytes (Splawski et al. 2004, 2005; Dick et al. 2016). Our data do not give an indication by which mechanism neuronal AP is prolonged since rise times and rates were affected similarly to fall times and rates. We found no difference in spike frequency adaptation in TS2-neo compared to WT mouse pyramidal cells.

The TS2 mutation has recently been found to prolong the AP duration in adrenal chromaffin cells (Calorio et al. 2019). Chromaffin cells have APs that are largely underpinned by sodium and potassium currents, similar to those which govern APs in neurons. In the chromaffin cell, the TS2 mutation causes a reduction in Na_V_1 channel density, which slows the rate of depolarization of the AP, resulting in a prolonged AP (Calorio et al. 2019). Ca_V_1.2 is known to impact gene transcription and ion channel trafficking within the neuronal membrane (Tian et al. 2014; Servili et al. 2020).

It is therefore more likely that the prolonged neuronal AP is caused by one or both of either a direct result of prolonged depolarizing currents through the mutant Ca_V_1.2 channel or a mutation-related impact on the Na_V_1 channel density in the neuronal membrane.

### Abnormal CSF

Neurons from TS2-neo mice were less likely to respond to visual stimuli of low SF but more likely to respond to visual stimuli of mid-to-high SF compared to WT mice. These results point to a basic visual processing abnormality in TS2-neo mice.

Three TS2 patients reported in the literature have been found to have abnormalities in eye movement and function, with a comorbid neurological disorder (Splawski et al. 2005; Walsh et al. 2018). While abnormalities in visual perception have not been reported in TS2 patients, our finding of abnormal visual function in the TS2-neo mouse suggests that the TS2 mutation, and perhaps TS mutations in general, may be associated with abnormalities in visual processing. This hypothesis is supported by a case report from a patient with atypical TS who exhibited severe visual dysfunction (Gillis et al. 2012).

It is possible that TS mutations result more generally in abnormalities in sensory perception. A study by Rendall et al. (2017) found that TS2-neo mice had a superior ability to respond to silent gaps within auditory stimuli compared to WT mice. It should be noted that TS is commonly associated with ASD: ~80% of TS patients meet the diagnostic criteria for ASD (Splawski et al. 2004). ASD often presents with abnormal sensory processing symptoms across all sensory modalities (Marco et al. 2011; Robertson and Baron-Cohen 2017). ASD patients have been found to be superior to neurotypical subjects in processing high-SF visual stimuli (Vlamings et al. 2010; Kéïta et al. 2014). Kéïta et al. (2014) found that ASD patients have an enhanced contrast sensitivity for simple visual grating stimuli of high SF.

As such, the TS2-neo mouse model of TS has also been considered as a mouse model of ASD of a specific known genetic cause (Bader et al. 2011b; Bett et al. 2012; Rendall et al. 2017). The superior auditory processing ability in TS2-neo mice found by Rendall et al. (2017) is similar to those previously reported in ASD (Bertone et al. 2005; Mottron et al. 2006). Cheng et al. (2020) found that the inbred BTBR mouse model of idiopathic ASD had higher contrast sensitivity to mid-to-high-SF visual stimuli compared to WT mice. In this study, we found that the TS2-neo mouse model had increased contrast sensitivity to visual stimuli of mid-to-high SFs but not at the highest SF tested (0.512 cpd). It is not clear yet how increased AP width might lead to an increase in sensitivity to visual stimuli of higher SF, but it is feasible that wider APs affect the temporal integration of bursts of APs elicited by grating stimuli, resulting in a stronger response to stimuli that in WT animals remain subthreshold.

Overall, our finding that TS2-neo mice have an abnormal CSF was similar to that found in ASD patients by Kéïta et al. (2014) and in the BTBR mouse model of ASD by Cheng et al. (2020). This suggests that the TS2-neo mouse has basic visual processing abnormalities which resemble those seen in ASD.

### Increased PV+ cell density

Inhibitory interneuron dysfunction is thought to be involved in the pathology of ASD (Hussman 2001) and epilepsy (Magloire et al. 2019), two common neurological phenotypes of TS. The PV+ cell number has been found to be abnormal in various areas of the brain in people with ASD by postmortem histological study (Lawrence et al. 2010; Zikopoulos and Barbas 2013; Ariza et al. 2016; Hashemi et al. 2017). Moreover, the gene coding for PV is the most strongly downregulated in the ASD brain as compared to the brain of neurotypical subjects, as found by postmortem transcriptome analysis (Schwede et al. 2018). This evidence has led to the PV hypothesis of ASD (Filice et al. 2020). A similar theory suggests that abnormal PV+ cell activity may result in epileptiform neural activity in the brain (Magloire et al. 2019). The evidence from this study supports the hypothesis that the ASD-related classical TS2 mutation results in abnormalities in PV+ cell density in the adult mouse brain.

Abnormalities in interneuron migration have been demonstrated in the developing brain of TS2-neo mice (Horigane et al. 2020). Beyond that, TS-related abnormalities in the development and density of PV+ interneurons have not previously been explored in animal models. However, PV+ cell number abnormalities have been reported for various brain areas in mouse models of ASD (Tripathi et al. 2009; Allegra et al. 2014; Lauber et al. 2016; Yang et al. 2021; Paterno et al. 2021b; Kourdougli et al. 2023). Specifically, the PV+ cell number has been found to be increased by 20% in V1 in the *En2* KO mouse model of ASD as compared to WT (Allegra et al. 2014); the *En2* gene encodes the homeobox protein engrailed-2, which is involved in embryonic development and is implicated in ASD (Benayed et al. 2009). Our study supports this finding; however, the sample size was relatively small, and further studies are needed to conclusively demonstrate an effect of classical TS mutations on PV+ cell development, number, and function in the mouse brain.

How might an increase in PV+ cell density affect neuronal CSFs? Visual gain control is modulated by different classes of GABAergic interneurons, including PV+ cells and somatostatin-positive and vasopressin-positive cells (Pakan et al. 2016). A higher density of PV+ cells might change the local balance of excitation and inhibition. Increased surround inhibition could result in a smaller excitatory receptive field center and therefore higher contrast sensitivity at higher SFs (Sengpiel et al. 1997).

## Conclusion

In summary, our results show that classical TS mutations (i) cause altered potential firing in principal visual cortical neurons, (ii) affect PV+ interneuron development and distribution in the visual cortex, and (iii) result in abnormalities in basic sensory processing within the visual domain. Study of the TS2-neo mouse model of TS is useful for gaining a better understanding of the neurophysiology of TS.

## Supplementary Material

Mouse_Model_of_Timothy_Syndrome_Supplementary_Material_final_bhaf162

## Data Availability

Data are openly available in the Cardiff University Research Data Repository at https://doi.org/10.17035/cardiff.29278625.
