# Supplementary material for: Disruptions in primary visual cortex physiology and function in a mouse model of Timothy syndrome
Source: Cereb Cortex. 2025 Jun 27;35(6):bhaf162. doi: 10.1093/cercor/bhaf162 (PMC12203796; doi:10.1093/cercor/bhaf162)
Supplement: Mouse_Model_of_Timothy_Syndrome_Supplementary_Material_final_bhaf162 [file mouse_model_of_timothy_syndrome_supplementary_material_final_bhaf162.docx]

Supplementary Material

Disruptions in Primary Visual Cortex Physiology and Function in a Mouse Model of Timothy Syndrome

Rosie Craddock^1,2^, Cezar M. Tigaret^2*^ and Frank Sengpiel^1,2*^

^1^School of Biosciences, Cardiff University, Museum Avenue, Cardiff CF10 3AX, UK

^2^Neuroscience and Mental Health Innovation Institute, Maindy Road, Cardiff CF24 4HQ, UK

*Corresponding authors

# Addresses: Frank Sengpiel: School of Biosciences, Cardiff University, Museum Avenue, Cardiff CF10 3AX, UK; [sengpielf@cardiff.ac.uk](mailto:sengpielf@cardiff.ac.uk)

Cezar M. Tigaret: Neuroscience and Mental Health Innovation Institute, Maindy Road, Cardiff CF24 4HQ, UK; [tigaretc@cardiff.ac.uk](mailto:tigaretc@cardiff.ac.uk)

**Supplementary Methods**

*Passive membrane properties*

The voltage trace corresponding to the -50 pA current step was used to measure passive membrane properties of the cell as well as voltage rebound and voltage sag. The passive membrane properties measured were membrane input resistance, capacitance, and the membrane time constant. The methods used to obtain these values were based on those reported by (Tamagnini et al. 2015). An illustration of how these measurements were obtained are shown in Supplementary Figure 1. Resting membrane potential was taken as the membrane potential of the cell measured 100 ms before the onset of the hyperpolarising current. This was typically -70 mV, and was not based on empirical measurement.

Rheobase was measured empirically as the minimum input current required to evoke an AP for the cell, measured in pA. No mathematical calculation was used to estimate rheobase to a higher degree of accuracy. The maximum instantaneous firing frequency of each cell was measured as the reciprocal of the minimum measured time between any two spikes for any input current to the cell. This was measured in Hz. Minimum AP onset latency was measured as the minimum time between stimulus onset and AP firing (taken from AP threshold). Minimum AP onset latency was measured in ms.

*Detection of cells in two-photon imaging*

Cell fluorescence traces were smoothed using a 100-point moving window and were down sampled 5 times. The mean fluorescence of the cell at the baseline period for a given stimulus was compared to that for the period during which each stimulus was shown. Cells which had a significantly higher fluorescence during the stimulus presentation as compared to the baseline period for stimuli of SFs shown at either 100% or 50% contrast were deemed to be visually responsive. The criteria were that the fluorescence had to be significantly higher during the stimulus as compared to the baseline period by Wilcoxon Signed Rank testing (threshold value = 0.01) with the average fluorescence being 30% higher for the stimulus as compared to the baseline period. Cells deemed to be visually responsive (to stimuli of any SF) were further tested to find which SFs they responded to, and the minimum contrast at which they responded to for that SF. Again, this involved Wilcoxon Signed Rank testing (threshold value = 0.01) and a 30% increase in fluorescence between the stimulus presentation and baseline periods. Data processing codes were written in MATLAB 2022a and are available on GitHub.

*Contrast response functions of visually responsive neurons*

Contrast sensitivity was classified in one of 7 categories for each of the 7 SFs (ordered from high to low), with both the independent (SF) and dependent (contrast sensitivity) variables being ordinal. A form of contrast sensitivity function (CSF) for cells from mice of each genotype was created by measuring the number of cells from mice of each genotype falling into each sensitivity category for stimuli of each SF. To assess how CSF varies by genotype, a cumulative link mixed model regression was completed where between-animal variance was taken as a random factor. The assumptions of the model were tested graphically and were found to be met. The goodness of fit of the model was tested using Cox and Snell method included in the nagelkerke function of the rcompanion package (Mangiafico 2023).

*Statistical analysis of PV+ cell density*

Analysis was completed in R. PV+ cell density data were visualised to assess distribution. A linear mixed model was used to explore how genotype impacted PV+ cell density in V1 while controlling for between-animal variance. This involved use of the lme4 package in R (Bates et al. 2015). Homoscedasticity was assessed visually by assessing equality of residuals across the fitted values of the model by eye, while normal distribution of the model residuals was tested using the Shapiro-Wilk method. The model was deemed to meet assumptions of homoscedasticity (not shown) and of normally distributed residuals (Shapiro-Wilk: W = 0.99, P = 0.23). The goodness of fit of the model was tested by estimating pseudo R^2^ values which were obtained using the MuMIn package in R (Barton 2024) which uses methods based on those of (Nakagawa and Schielzeth 2013). The effect of genotype on PV+ density in V1 was assessed by comparing the statistical model described above against a null model—which accounted for between-animal variance but did not model PV+ cell density by genotype—using analysis of variance. Effect size was estimated from the model directly.

**References**

Barton K. 2024. CRAN - Package MuMIn.

Bates D, Mächler M, Bolker BM, Walker SC. 2015. Fitting Linear Mixed-Effects Models Using lme4. Journal of Statistical Software. 67:1-48.

Mangiafico S. 2023. Functions to Support Extension Education Program Evaluation [R package rcompanion version 2.4.34].

Nakagawa S, Schielzeth H. 2013. A general and simple method for obtaining R2 from generalized linear mixed-effects models. Methods in Ecology and Evolution. 4:133-142.

Paşca SP, Portmann T, Voineagu I, Yazawa M, Shcheglovitov A, Paşca AM, Cord B, Palmer TD, Chikahisa S, Nishino S, Bernstein JA, Hallmayer J, Geschwind DH, Dolmetsch RE. 2011. Using iPSC-derived neurons to uncover cellular phenotypes associated with Timothy syndrome. Nat Med. 17:1657-1662.

Tamagnini F, Novelia J, Kerrigan TL, Brown JT, Tsaneva-Atanasova K, Randall AD. 2015. Altered intrinsic excitability of hippocampal CA1 pyramidal neurons in aged PDAPP mice. Frontiers in Cellular Neuroscience. 9:372-372.

**Supplementary figures**


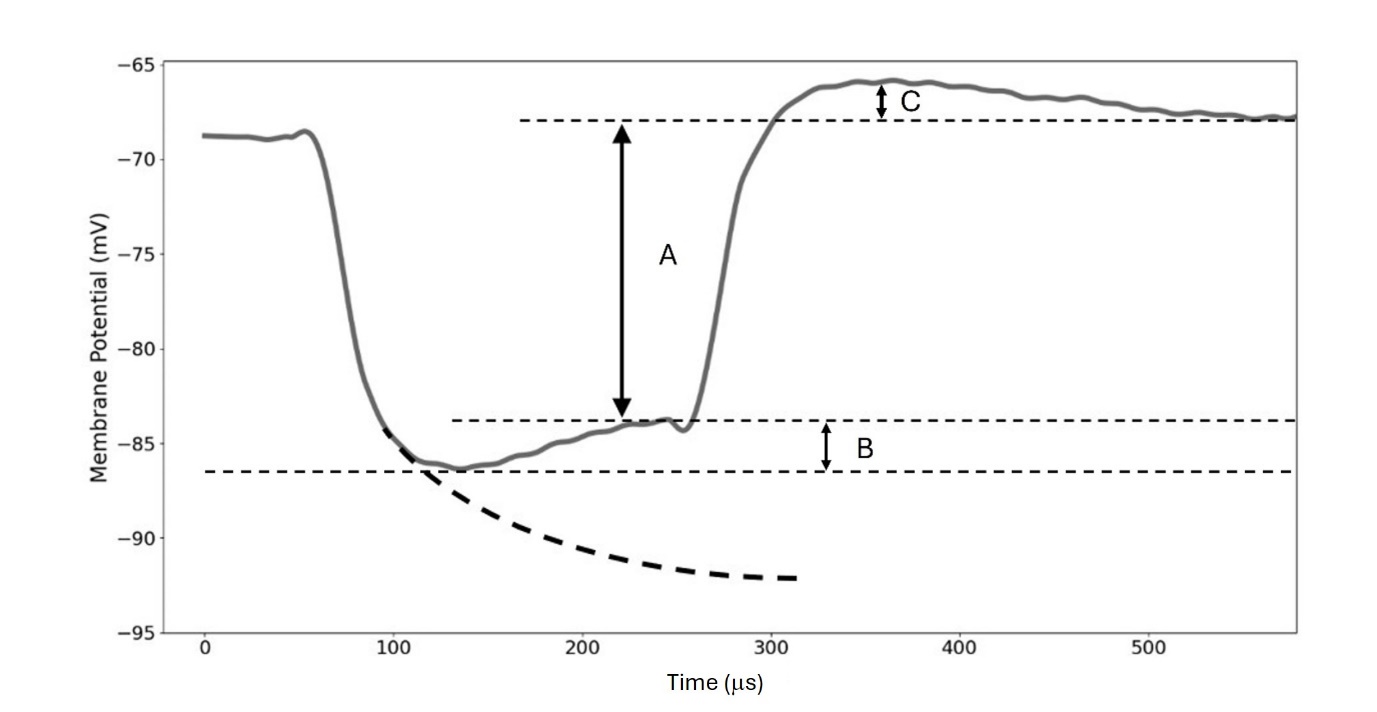


**Supplementary Figure 1**: Schematic showing the voltage trace corresponding to a -50 pA current injection and the measurements taken to obtain values for passive membrane properties, voltage sag and membrane potential rebound. The voltage difference, A, divided by the input current (-50 pA) is taken as the input resistance (MΩ). The measurement B was used to give the voltage sag (mV). The measurement C was used to obtain the membrane potential rebound (mV). An exponential fit of the curve of the depolarisation (fitted based on methods of Tamagnini et al. (2015), indicated by the dashed line) was used to obtain a measure of the membrane time constant, τ (ms). Membrane capacitance was taken as τ/input resistance (pF).


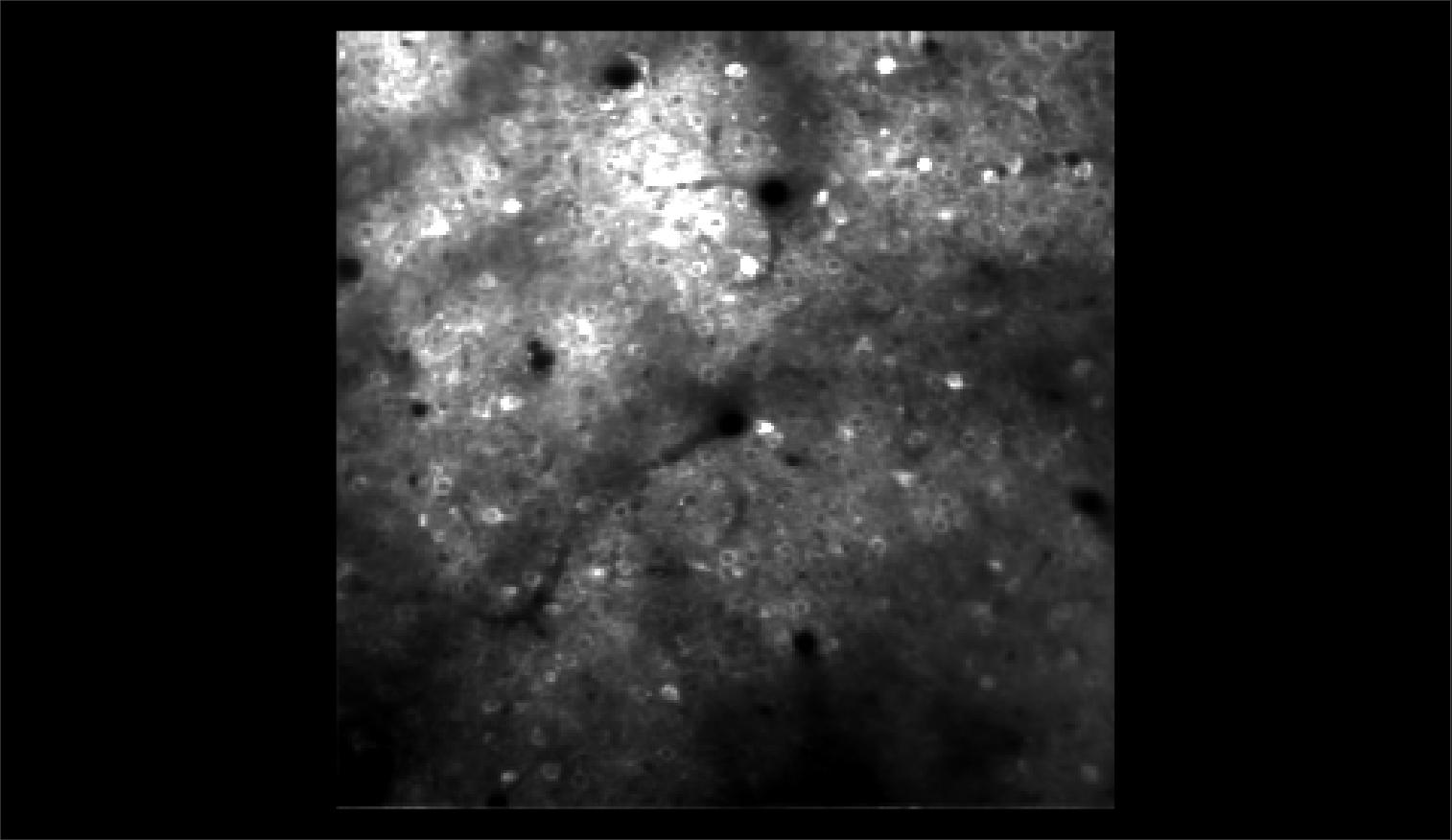


**Supplementary Figure 2**: GCaMP6f-labelled neurons in V1 of a TS2-neo mouse responding to a grating stimulus of low SF (0.014 cpd) and high contrast (100%). Scale bar, 100 μm.
